# Data on the oral CRTh2 antagonist QAW039 (fevipiprant) in patients with uncontrolled allergic asthma

**DOI:** 10.1016/j.dib.2016.08.039

**Published:** 2016-08-29

**Authors:** Veit J. Erpenbeck, Todor A. Popov, David Miller, Steven F. Weinstein, Sheldon Spector, Baldur Magnusson, Wande Osuntokun, Paul Goldsmith, Markus Weiss, Jutta Beier

**Affiliations:** a Novartis Pharma AG, Basel, Switzerland; b Clinic of Allergy & Asthma, Medical University Sofia, Sofia, Bulgaria; c NEMRA, North Dartmouth, MA, United States; d Allergy & Immunology, Allergy and Asthma Specialists Medical Group and Research Center, CA, United States; e Allergy & Asthma, California Allergy and Asthma Medical Group Inc, CA, United States; f Novartis Pharmaceuticals, Horsham, United Kingdom; g INSAF, Respiratory Research Institute GmbH, Wiesbaden, Germany

## Abstract

This article contains data on clinical endpoints (Peak Flow Expiratory Rate, fractional exhaled nitric oxide and total IgE serum levels) and plasma pharmacokinetic parameters concerning the use of the oral CRTh2 antagonist QAW039 (fevipiprant) in mild to moderate asthma patients. Information on experimental design and methods on how this data was obtained is also described. Further interpretation and discussion of this data can be found in the article “The oral CRTh2 antagonist QAW039 (fevipiprant): a phase II study in uncontrolled allergic asthma” (Erpenbeck et al., in press) [1].

**Specifications Table**

**Table:** 

Subject area	*Randomized clinical trial, medicine*
More specific subject area	*Allergic asthma*
Type of data	*Tables*
How data was acquired	*Clinical study protocol, spirometry, Fractional exhaled nitric oxide, total serum IgE, rescue medication use and pharmacokinetic evaluation*
Data format	*Analyzed*
Experimental factors	*Randomized clinical trial in uncontrolled allergic asthma*
Experimental features	*The efficacy of QAW039 in patients with mild to moderate persistent allergic asthma was assessed using Peak Flow Expiratory Rate (PEFR), Fractional Exhaled Nitric Oxide (FeNO), total serum IgE levels and the use of rescue medication over 28 days. Data was compared with patients who were randomized to receive placebo. A subgroup analysis of patients with a FEV*_*1*_*(% predicted) <70% was carried out evaluating PEFR and FeNO. Pharmacokinetic profiling of QAW039 was carried out using LC–MS/MS method*
Data source location	*Basel, Switzerland.*
Data accessibility	*Data with this article*

**Value of the data**

• The data presented on clinical endpoints are important for studies performed for clinical development of drugs in asthma to determine aspects of clinical and mechanistic efficacy.
• The pharmacokinetic data from this study help to describe the compound׳s properties and support its suitability for once daily dosing.
• Uncontrolled allergic asthma patients with a FEV_1_ (% predicted) <70% might benefit from once daily QAW039 treatment, but further data is required

## 1 Data

A detailed inclusion and exclusion criteria for the clinical trial can be found in Table 1. Patients taking inhaled corticosteroids and/or LABA/other asthma medications entered a weaning period whereby a stepwise tapering of their asthma medication occurred every 7 days (Figure 1 in [1]). Peak Flow Expiratory Rate in the morning and evening for the PD analysis set over 28 days are highlighted in Table 2.

Statistical analysis of fractional exhaled nitrous oxide and total serum IgE over 28 days in the PD analysis set and the subgroup of patients with a FEV_1_ (% predicted) <70% is highlighted in Tables 3 and 4, respectively.

Rescue medication (salbutamol/albuterol) use over 28 days in the PD analysis set is displayed in Table 5.

In Table 6, results from the plasma PK parameters of QAW039 on Days 1 and 28 from the PK analysis set are highlighted.

## 2 Experimental design, materials and methods

### 2.1 Weaning period, patient randomization and drug administration

A weaning period was carried out in using a 3 step method prior to randomization to either treatment arm. At Step 1, all non-ICS asthma medication (e.g. LABA) was stopped, with SABA rescue medication (salbutamol/albuterol) for on demand administration. At Step 2/3, ICS doses were reduced from a medium daily dose to a low daily dose and/or from a low daily dose to a complete withdrawal of ICS. Postweaning, patients were randomized to either QAW039 500 mg or placebo treatments. Both treatments were administered to patients in a double-blind fashion (both the patient and investigator remained blinded to the treatment allocation). The appearance of both treatments was identical to maintain blinding. Randomization data were kept strictly confidential, and were accessible only to authorized personnel until database lock.

Treatments were administered with approximately 200 mL of water. Patients who were randomized were dosed in the morning (between 07:00 and 10:00). Patients received treatments at their respective clinical trial site on Days 1, 7, 14, 21 and 28 which corresponded with time points for recording the data for the respective clinical endpoints (Peak expiratory flow rate, fractional exhaled nitric oxide, total serum IgE and pharmacokinetics).

### 2.2 Peak expiratory flow rate, fractional exhaled nitric oxide and total serum IgE

Peak expiratory flow rate (PEFR) was measured by the patients using a home spirometry device both in the in morning and evening over the course of the 28 day period. Time course of PEFR was plotted for morning and evening assessments, and analyzed using a repeated measures mixed effects model. The model included treatment and time as fixed factors, the treatment by time interaction terms, mean baseline PEFR values as a covariate, and patient as a random effect. A statistical analysis was also performed on the mean morning and evening PEFR values on Days 1, 2, 7, 14, 21 and 28.

Fractional exhaled nitric oxide (FeNO) has been successfully used to monitor anti-inflammatory treatment and was assessed using the NIOX MINO analyzer in line with the recommendations for standardized procedures for the online and offline measurement of exhaled lower respiratory nitric oxide and nasal nitric oxide in adults and children [2]. Blood samples (10 mL) were collected and serum obtained to determine levels of total IgE. Quantification of IgE was part of the standard laboratory practice.

### 2.3 Pharmacokinetic evaluation

All patients who received QAW039 or placebo had blood and urine samples collected for pharmacokinetic (PK) analysis. Blood samples (2×3 mL) for pharmacokinetic evaluation were collected from a forearm vein (direct venipuncture or from an indwelling cannula) into vacuum polypropylene tubes or monovettes containing EDTA as anticoagulant at each time point. Within 10 min of collection, blood samples were centrifuged at 3000×*g* for 15 min between 0 and 4 °C. Following centrifugation, 1 mL of the plasma was transferred into a polypropylene screw cap tube containing lactic acid solution (10 μL of lactic acid at 70% per mL of plasma), and stored at ≤−70 °C.

Urine samples were collected on Day 1 (pre-dose) and over a 24 h period on Day 28. During each sampling interval the urine portions were pooled in a polypropylene container and stored either on ice or in a refrigerator at 0–4 °C. Immediately after the end of the collection interval, 2×5 mL aliquots of urine were removed from the pooled sample and placed into polypropylene tubes and frozen at ≤−70 °C within 15 min of the collection. Prior to this, 5 mL aliquot of urine per time period was then transferred into polypropylene tubes containing lactic acid solution (10 μL of lactic acid at 70% per mL urine).

Concentrations in plasma and urine of QAW039 and the glucuronide metabolite CCN362 were determined by a validated LC–MS/MS method with a LLOQ of approximately 1 ng/mL for QAW039 and 2 ng/mL for CCN362.

## Figures and Tables

**Table 1:** Detailed inclusion and exclusion criteria.

**Inclusion criteria**
At screening:
1. Written informed consent must be obtained before any study specific assessments are performed.
2. Male and post-menopausal female asthma patients 18–65 years of age inclusive.
3. Patients with a medical history of mild-to-moderate persistent allergic asthma, diagnosed according to (GINA 2009) guidelines.
4. A positive skin prick test to aeroallergens, such as tree, grass, pollen, pet dander, house dust mite or cockroach antigens. In addition, any allergens specific to the country/locality can be included. Historic skin prick test results within the 12 months prior to screening are acceptable (supported with clinical documentation).
5. Women must be postmenopausal or surgically sterilized at the time of participation.
• Postmenopausal females must have 12 months of natural (spontaneous) amenorrhea prior to dosing OR 6 months of spontaneous amenorrhea with serum FSH levels >40 IU/L at screening. • Female patients who reported surgical sterilization must have had the procedure at least 6 months prior to initial dosing. • Surgical sterilization procedures should be supported with clinical documentation made available to the sponsor and noted in the Relevant Medical History/Current Medical Conditions section of the eCRF. • All female patients must have negative pregnancy test results at screening and at baseline.
6. Male patients must agree to use two acceptable methods of contraception, (e.g. spermicidal gel plus condom) for the entire duration of the study and up to the study completion visit, and refrain from fathering a child in the three months following the last study drug administration Periodic abstinence and withdrawal are not acceptable methods of contraception.
7. Patients must weigh at least 45 kg to participate in the study, and must have a body mass index (BMI) of >17 kg/m^2^.
8. Patients must be able to communicate well with the Investigator, so they can understand and comply with the requirements of the study.
9. Patients must demonstrate an increase of ≥12% AND 200 mL in FEV_1_ over their prebronchodilator value within 30 min after inhaling a total of 400/360 μg of salbutamol/albuterol (the reversibility test). Reversibility will have to be demonstrated after an appropriate washout period of at least 6 h for a short-acting β2-agonist. The administration of salbutamol/albuterol for the reversibility test is to be within 30 min after pre-bronchodilator spirometry. Reversibility has to be determined at screening or during the weaning period (up until visit 5). Eligibility to continue the study will be checked at baseline before randomization. Inclusion criteria 1–9 from screening must be reviewed at baseline. Additional inclusion criteria applied:
10. Patients must have had FEV_1_ of ≥60% and ≤85% of the predicted normal value for the patient when LABA and steroid-weaned. This criterion for FEV_1_ will have to be demonstrated after a washout period of at least 6 h during which no short acting β2- agonist has been inhaled.
11. Patients must be symptomatic after weaning of their asthma medication which will be assessed by a mean rescue medication usage of at least 1 puff per day (average of 7 days prior to baseline visit).
12. Patients must have an ACQ score of at least 1.5 at baseline.
**Exclusion criteria**
1. Use of other investigational drugs at the time of enrollment, or within 30 days or 5 half-lives of enrollment, whichever is longer; or longer if required by local regulations, and for any other limitation of participation in an investigational trial based on local regulations.
2. History of hypersensitivity to any of the study drugs or to drugs of similar chemical classes (CRTh2 antagonists).
3. A history of clinically significant ECG abnormalities or recent history of autonomic dysfunction (e.g. recurrent episodes of fainting, palpitations etc.).
4. History of malignancy of any organ system (other than localized basal cell carcinoma of the skin), treated or untreated, within the past 5 years, regardless of whether there is evidence of local recurrence or metastases.
5. Pregnant or nursing (lactating) women.
6. Women of child-bearing potential.
7. Smokers defined as history of smoking in the previous 6 months or a smoking history of more than 10 pack-years, a pack-year being defined as smoking the equivalent of 20 cigarettes – a pack – every day for the period of 1 year.
8. Patients with severe persistent asthma according to GINA 2009 guidelines.
9. Patients treated with systemic or high-dose ICS, sustained release theophylline or omalizumab.
10. History of life-threatening asthma, defined as an asthma episode that required intubation and/or was associated with hypercapnia, respiratory arrest and/or hypoxic seizures. History of asthma exacerbation in the past 6 months that required hospitalization or emergency unit visit. Use of parenteral steroids within 6 months of screening.
11. Use of a biologic (e.g. monoclonal antibodies) agent for the treatment of asthma in the past 6 months.
12. Any disease or illness, other than asthma, that may require the use of systemic corticosteroids during the study period.
13. Any occupational exposure to allergens/irritants that may have a potential to worsen the asthma symptoms during the trial.
14. Respiratory tract infection and/or exacerbation of asthma within 4 weeks prior to the first dose of study medication. Patients with other serious underlying diseases (i.e. tuberculosis, bronchiectasis, pulmonary fibrosis, pulmonary hypertension, emphysema, chronic bronchitis, α-1-antitrypsin deficiency). Note: When patient has upper respiratory signs and symptoms due to common cold post-screening and prior to dosing, baseline evaluation should be delayed until symptoms resolve.
15. Use of any prescription drugs;
• herbal supplements, within 4 weeks prior to initial dosing, and/or • over-the-counter (OTC) medication, • dietary supplements (vitamins included) within 2 weeks prior to initial dosing other than short-acting inhaled beta-agonists • paracetamol/acetaminophen for the treatment of minor ailments e.g. headache from 48 h before the first dose until the end of study visit is acceptable, but must be documented in the concomitant medications/significant non-drug therapies page of the clinical report form. • Patients requiring stable use of medications for concomitant conditions such as hypertension, hypercholesterolemia, diabetes, thyroid gland disease or hormone replacement therapy can be included allowed/prohibited.
16. Donation or loss of 400 ml or more of blood within 8 weeks prior to initial dosing, or longer if required by local regulation. Hemoglobin levels below normal range at screening.
17. Significant illness within 2 weeks prior to initial dosing.
18. Any surgical or medical condition which might significantly alter the absorption, distribution, metabolism, or excretion of drugs, or which may jeopardize the patient in case of participation in the study. The Investigator should make this determination in consideration of the patients medical history and/or clinical or laboratory evidence of any of the following:
• Inflammatory bowel disease, ulcers, gastrointestinal or rectal bleeding • Major gastrointestinal tract surgery such as gastrectomy, gastroenterostomy, or bowel resection • Pancreatic injury or pancreatitis • Liver disease or liver injury as indicated by abnormal liver function tests such as SGOT (AST), SGPT (ALT), γ-GGT, alkaline phosphatase,

**Table 2:** Statistical analysis of Peak Flow Expiratory Rate in the morning and evening in the PD analysis set.

Morning	QAW039	Placebo	Treatment difference QAW039-placebo
Day			
1	389.6 (377.9, 401.4)	382.8 (371.1, 394.4)	6.9 (−9.7, 23.4)^NS^
2	395.0 (382.9, 407.1)	398.8 (387.5, 410.0)	−3.8 (−20.3, 12.8)^NS^
7	394.4 (382.9, 405.9)	388.6 (377.2, 400.0)	5.8 (−10.4, 22.0)^NS^
14	393.3 (381.4, 405.2)	389.8 (378.4, 401.2)	3.5 (−13.0, 20.0)^NS^
21	394.1 (381.9, 406.3)	391.5 (379.9, 403.1)	2.6 (−14.3, 19.5)^NS^
28	378.6 (366.3, 391.0)	382.8 (371.3, 394.4)	−4.2 (−21.1, 12.7)^NS^


Evening	QAW039	Placebo	Treatment difference QAW039-placebo
Day			

1	395.8 (382.4, 409.2)	390.8 (378.6, 403.0)	4.9 (−13.1, 23.0)^NS^
2	420.9 (407.7, 434.1)	407.5 (394.4, 420.7)	13.4 (−5.3, 32.0)^NS^
7	402.9 (388.9, 416.9)	394.0 (381.6, 406.3)	8.9 (−9.7, 27.6)^NS^
14	414.6 (400.8, 428.3)	382.9 (370.2, 395.5)	31.7 (13.1, 50.3)⁎⁎
21	409.5 (395.2, 423.7)	394.4 (380.9, 407.9)	15.1 (−4.5, 34.7)^NS^
28	391.0 (376.5, 405.5)	379.9 (367.4, 392.4)	11.1 (−8.0, 30.2)^NS^

Data are least squares mean, 90% CI.

NS, not significant.

⁎⁎ *P*<0.01.

**Table 3:** Statistical analysis of fractional exhaled nitrous oxide in the PD analysis set and the FEV_1_ [% predicted] <70% group.

Placebo			
**PD analysis set**			
Day	QAW039	Placebo	Treatment difference QAW039-placebo

7	31.471 (29.539, 33.528)	35.059 (32.864, 37.400)	0.898 (0.820, 0.983)⁎
14	30.090 (27.917, 32.432)	33.768 (31.275, 36.459)	0.891 (0.800, 0.992)⁎
21	29.913 (27.744, 32.253)	35.016 (32.393, 37.852)	0.854 (0.767, 0.952)⁎⁎
28	31.521 (29.015, 34.244)	32.805 (30.104, 35.749)	0.961 (0.853, 1.083)^NS^
29	31.685 (28.940, 34.691)	34.331 (31.220, 37.752)	0.923 (0.809, 1.052)^NS^

**FEV**_**1**_**[% predicted] <70%**			
Day	QAW039	Placebo	Treatment difference QAW039 - placebo

7	37.912 (33.722, 42.623)	40.666 (36.448, 45.373)	0.932 (0.794, 1.095)^NS^
14	37.318 (32.514, 42.832)	38.538 (33.903, 43.806)	0.968 (0.802, 1.169)^NS^
21	36.641 (31.880, 42.113)	41.403 (36.307, 47.216)	0.885 (0.731, 1.072)^NS^
28	38.712 (33.388, 44.885)	40.753 (35.443, 46.858)	0.950 (0.775, 1.164)^NS^
29	37.698 (32.314, 43.979)	43.921 (37.845, 50.973)	0.858 (0.693, 1.064)^NS^

Data are least squares mean, 90% CI.

NS, not significant.

⁎ *P*<0.05.

⁎⁎ *P*<0.01.

**Table 4:** Statistical analysis of serum total IgE (μg/L) in the PD analysis set and the and FEV_1_ [% predicted] <70% group.

**PD analysis set**			
Day	QAW039	Placebo	Treatment difference QAW039-placebo
7	401.4 (392.0, 411.2)	406.1 (397.3, 415.0)	0.989 (0.957, 1.021)^NS^
14	399.8 (387.3, 412.8)	401.1 (389.4, 413.1)	0.997 (0.954, 1.041)^NS^
21	402.0 (388.5, 416.0)	402.4 (389.8, 415.4)	0.999 (0.953, 1.047)^NS^
28	403.7 (370.7, 439.5)	415.1 (383.8, 448.8)	0.973 (0.866, 1.092)^NS^

**FEV**_**1**_**[% predicted] <70%**			
Day	QAW039	Placebo	Treatment difference QAW039-placebo

7	349.7 (336.7, 363.3)	355.1 (344.9, 365.6)	0.985 (0.938, 1.034)^NS^
14	328.5 (310.9, 347.1)	355.3 (340.2, 371.1)	0.925 (0.862, 0.992) ⁎
21	327.8 (308.2, 348.7)	354.7 (337.6, 372.7)	0.924 (0.854, 1.001)^NS^
28	336.2 (303.3, 372.6)	340.4 (313.3, 369.9)	0.988 (0.865, 1.127)^NS^

Data are least squares mean, 90% CI.

NS, not significant.

⁎ *P*<0.05.

**Table 5:** Statistical analysis of salbutamol/albuterol usage in the PD analysis set.

Day	QAW039	Placebo	Treatment difference QAW039-placebo
1–7	2.056 (1.731, 2.441)	1.917 (1.621, 2.266)	1.072 (0.844, 1.363)^NS^
7–14	2.111 (1.795, 2.483)	2.047 (1.754, 2.390)	1.031 (0.824, 1.290)^NS^
14–21	1.929 (1.625, 2.291)	2.018 (1.725, 2.363)	0.956 (0.757, 1.207)^NS^
21–28	1.748 (1.468, 2.080)	1.903 (1.628, 2.224)	0.919 (0.727, 1.160)^NS^

Data are least squares mean, 90% CI.

NS, not significant.

**Table 6:** Summary statistics of plasma PK parameters of QAW039 on Days 1 and 28 (PK analysis set).

Day	Statistic	Tmax (h)^a^	Cmax (ng/mL)	AUCtau^b^ (h*ng/mL)	Cav (ng/mL)	Cmin (ng/mL)	Racc AUCtau^c^	Ratio Cmax^d^
1	*N*	73	73	73	73			
	Mean (SD)	1.08	3610 (1900)	12,000 (5500)	502 (229)	*NC*	*NC*	*NC*
	CV%	(0.47, 6.00)	52.6	45.7	45.7			
28	*N*	71	71	70	70	71	66	67
	Mean (SD)	1.13	3440 (1850)	14,300 (6090)	595 (254)	118 (104)	1.23 (0.271)	1.13 (0.719)
	CV%	(0.50, 4.05)	53.8	42.7	42.7	87.8	22.0	63.6

NC=not calculated.

a Median (min, max).

b tau=AUC0-24 h (Day1), AUC0-24 h (Day 28).

c Racc AUCtau=AUCtau Day 28/AUCtau Day1.

d Ratio Cmax=Cmax Day 28/Cmax Day 1.
