# Supplementary material for: Data on the oral CRTh2 antagonist QAW039 (fevipiprant) in patients with uncontrolled allergic asthma
Source: Data Brief. 2016 Aug 29;9:199–205. doi: 10.1016/j.dib.2016.08.039 (PMC5021787; doi:10.1016/j.dib.2016.08.039)
Supplement: Supplementary file 1 — Supplementary material [file mmc1.doc]

**Conflict of interests**

This trial was funded by Novartis Pharma AG, Basel Switzerland. **VJE**, **BM** and **MW** are employees of Novartis. **TAP** has nothing to declare. DM has nothing to declare. **SFW** reports grants from Novartis, grants from Pulmagen, grants from Teva, grants from GlaxoSmithKline, grants from Astra-Zeneca, grants from Mylan, grants from Roxane. **SS** reports stock and mutual funds in GlaxoSmithKline and Merck; has received grant support from TKL, Perrigo, Targacept, Genentech, Novartis, Sanofi-Aventis, AstraZeneca, GlaxoSmithKline, Amgen, Merck, Boehringer Ingelheim, and Sunovion; and is a speaker/moderator for the American College of Allergy, Asthma, and Immunology. **WO** reports that she was an employee of Novartis Pharmaceuticals, Horsham, United Kingdom at the time of this study and is currently an employee of Takeda Development Centre Europe, London, United Kingdom. **PG** reports he was an employee of Novartis Pharmaceuticals, Horsham, United Kingdom at the time of this study and is currently an employee of Takeda Pharmaceuticals Europe, London, United Kingdom. **JB** reports advisory board, lecture fees, compensation for conduct of clinical trials paid to insaf Respiratory Research Institute from Almirall Hermal, Novartis and AstraZeneca; compensation for conduct of clinical trials paid to INSAF Respiratory Research Institute from TEVA, Mundipharma, Sterna AG, Boehringer Ingelheim and GSK; and advisory board and/or lecture fees from Berlin Chemie, Menarini, Pohl Boskamp.
